# Ultrasensitive, Multiplexed Buoyant Sensor for Monitoring Cytokines in Biofluids

**DOI:** 10.1021/acs.nanolett.3c02516

**Published:** 2023-11-03

**Authors:** Heng Guo, Rohit Gupta, Dhavan Sharma, Elizabeth Zhanov, Connor Malone, Ravi Jada, Ying Liu, Mayank Garg, Srikanth Singamaneni, Feng Zhao, Limei Tian

**Affiliations:** † Department of Biomedical Engineering, Texas A&M University, College Station, Texas 77843, United States; ‡ Department of Mechanical Engineering and Materials Science, Institute of Materials Science and Engineering, Washington University in St. Louis, St. Louis, Missouri 63130, United States; § Center for Remote Health Technologies and Systems, Texas A&M University, College Station, Texas 77843, United States

**Keywords:** multiplexed biosensors, ultrasensitive protein detection, digital, immune biomarkers, cytokines

## Abstract

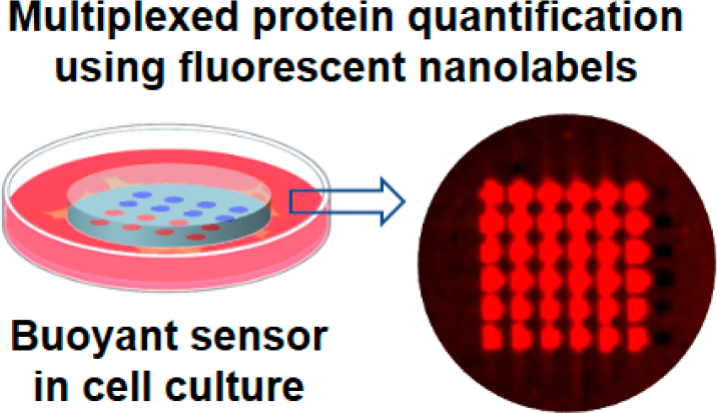

Multiplexed quantification of low-abundance protein biomarkers in complex biofluids is important for biomedical research and clinical diagnostics. However, in situ sampling without perturbing biological systems remains challenging. In this work, we report a buoyant biosensor that enables in situ monitoring of protein analytes at attomolar concentrations with a 15 min temporal resolution. The buoyant biosensor implemented with fluorescent nanolabels enabled the ultrasensitive and multiplexed detection and quantification of cytokines. Implementing the biosensor in a digital manner (i.e., counting the individual nanolabels) further improves the low detection limit. We demonstrate that the biosensor enables the detection and quantification of the time-varying concentrations of cytokines (e.g., IL-6 and TNF-α) in macrophage culture media without perturbing the live cells. The easy-to-apply biosensor with attomolar sensitivity and multiplexing capability can enable an in situ analysis of protein biomarkers in various biofluids and tissues to aid in understanding biological processes and diagnosing and treating diverse diseases.

Sensitive detection and quantification of various protein biomarkers in biological fluids and tissues is of fundamental importance in biomedical research and clinical diagnostics.^1,2^ Traditional methods such as enzyme-linked immunosorbent assay (ELISA), Western Blot, and mass spectrometry have enabled the discovery and investigation of proteins in biological samples.^3^ However, these techniques cannot provide an *in situ* analysis. For example, to monitor and quantify the protein concentration changes in cell culture media over time, sample replicates need to be prepared and characterized at specific time points throughout the duration of a study.^4−6^ The large variations between different batches of cell cultures can potentially mask the temporal concentration changes in the biomarkers of interest. These methods fail to provide an evaluation with high temporal resolution and statistical power, and they are also time-consuming and expensive, because of the need for cell culture replicates. In-situ monitoring and quantification of protein biomarkers in these complex biofluids without perturbing live cells remain challenging.

Cytokines are small (6–70 kDa) proteins that affect almost every biological process, ranging from embryonic development to disease pathogenesis and aging.^7^ Understanding the dynamics of cytokine secretion by immune cells over time is important for revealing the functionality of the cytokine in an immune response. Various biosensors based on protein arrays and bead-based immunoassays have been reported to enable simultaneous quantification of multiple cytokines with small sample volumes.^8^ For example, commercial fluorescent bead-based technology, including Luminex-based or cytometric bead array technologies, requires a sample volume of 5–100 μL.^9−13^ The low detection limit for most high-sensitivity immunoassays and biosensors is typically in the range of 0.1–1 pg/mL. However, the dynamically secreted concentration of cytokines in biofluids is often below 0.1 pg/mL and therefore not quantifiable. Several recent amplification technologies enabled the detection in the low fg/mL range through plasmon-enhanced fluorescence,^14^ biobarcode amplification,^15^ photonic-plasmonic coupling,^16^ electrochemiluminescence,^17^ or molecular on-bead signal amplification.^18^ Such low detection limits were achieved with the sample incubation of 1 h or longer and typical sample processing with reagent diluents. The long sampling time limits the temporal resolution in continuous monitoring. Therefore, multiplexed biosensors to quantify low concentrations of cytokines in unprocessed biofluids with a short sampling time remain as an unmet need.

In this work, we report a buoyant (i.e., floating) sensor that enables multiplexed detection and quantification of cytokines in culture media with an attomolar detection limit. The buoyant sensor operates by resting on the surface of the culture media for a short duration of 15 min during sampling, thereby not perturbing the live cells in the culture. The sensors introduced at different times can quantify the temporal changes in the concentrations of cytokines secreted by the same cell populations. For a proof-of-concept demonstration, human interleukin 6 (IL-6) and tumor necrosis factor-α (TNF-α) served as model protein analytes. The short sampling time allows for in situ monitoring of IL-6 and TNF-α in the culture media with high temporal resolution. The low limits of detection for IL-6 and TNF-α were found to be 3.5 fg/mL (148 aM) and 21 fg/mL (808 aM), respectively, with multiplexed biosensors. We harnessed ultrabright fluorescent nanolabels that can be counted with a standard fluorescent microscope to further improve the sensitivity, which has not been reported previously. With digital counting, the low limit of detection for IL-6 was further reduced to 14 ag/mL (0.6 aM), which is the lowest reported concentration achieved by nanobiosensors.

The buoyant sensor comprises a poly(dimethylsiloxane) (PDMS) disk, a flexible polystyrene (PS) film, and a capture antibody microarray printed on the PS film (Figure 1a). PDMS is biocompatible and hydrophobic and has a low density (0.97 kg/m^3^), allowing the sensor to float on the surface of cell culture media without disturbing the live cells. The PS surface was treated with Gamma irradiation to increase capture antibody binding.^19^ To demonstrate the multiplexed capability, we chose human IL-6 and TNF-α as model inflammation biomarkers. Dynamic changes in the concentrations of these cytokines in various biofluids, including cell culture media,^5,20^ interstitial fluid,^21,22^ and blood,^23,24^ are associated with various biological and physiological conditions. The IL-6 and TNF-α secretions are extremely dynamic, and the half-lives of IL-6 and TNF-α are 15.5 h and 18.2 min, respectively.^8^ To detect and quantify these two cytokines simultaneously, we printed the IL-6 and TNF-α capture antibody microarrays on the PS surface in each sensor. Subsequently, the sensor was set afloat on the culture media surface for 15 min to capture IL-6 and TNF-α, followed by the plasmon-enhanced fluorescence-linked immunosorbent assay (pFLISA). The sensors introduced at different times quantified the temporal changes in the cytokine concentration from the same culture. After capturing the cytokine targets during floating, the sensors were incubated with IL-6 and TNF-α detection antibodies and then fluorescent nanolabels. These ultrabright fluorescent nanolabels, called plasmonic-fluors (PFs), are comprised of a plasmonic nanostructure coated with fluorescent dye IR650 and streptavidin (PF-650). Previous studies show that PFs are nearly 10,000-fold brighter compared to the corresponding conventional fluorophores.^14,25^

**Figure 1:**
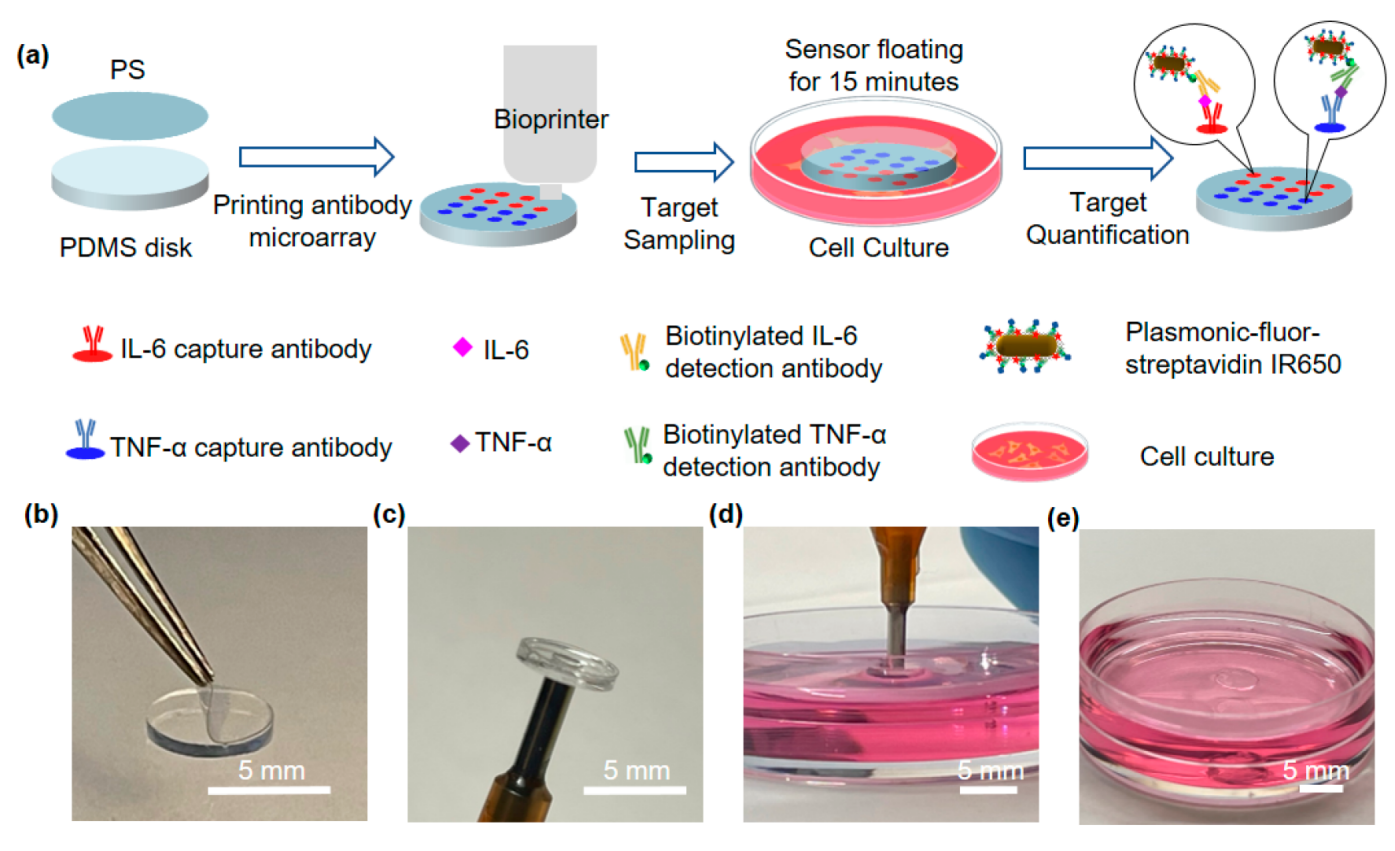
Nonperturbing buoyant sensor concept illustration. (a) Schematic illustration of fabrication and application of the buoyant sensor for monitoring multiple protein analytes. Optical images of (b) assembling, (c) handling, and (d, e) deploying the buoyant sensor.

Figure 1b shows a sensor with a PS film laminated on the PDMS disk. The PDMS disk is 5 mm in diameter and 600 μm in thickness, and the PS film has the same diameter and a thickness of 30 μm, which can fit into a 96-well microplate. Both components are optically transparent and ideal to use in pFLISA. A picosecond laser cutter was used to cut the PS film. It is important that the flexible PS film seamlessly adheres to the PDMS surface to eliminate undesired fluorescence backgrounds and ensure reliable sensor performance (Figure S1b). In contrast, the PS circular films cut by a CO_2_ laser exhibited material degradation and plastic deformation at the edge, resulting in a gap between the PS and the PDMS surface (Figure S1a). Such a nonconformal interface results in unwanted nonspecific binding and fluorescence backgrounds (Figure S1c). The sensor can be easily picked up by a vacuum pick-up tool and placed on the surface of culture media without disturbing the live cells (Figure 1c–e).

We designed and printed antibody microarrays to achieve the multiplexed quantification of cytokine targets. Droplet jetting is a powerful printing approach to pattern different functional materials on various substrates.^26,27^Figure 2a shows a microdot array design with asymmetric spatial control markers to determine the location of each microdot for quantification. We printed the IL-6 capture antibody microarray on the PS surface to characterize and optimize the sensor performance. Antibody droplets of 3 nL yield individual microdots with a diameter of 220 μm, and the center-to-center distance between microdots is 500 μm (Figure 1b). Biotinylated antibodies were printed on the control spots, which bind to PF nanolabels conjugated with streptavidin. After printing, the PS surface was blocked with bovine serum albumin (BSA) for 1 h and then exposed to varying concentrations of IL-6 in reagent diluent, followed by binding of biotinylated detection antibody to the captured IL-6 and PF labeling. The PF nanolabels were synthesized following the previous protocols.^14,25^ Silver-coated gold nanorods (AuNRs@Ag) with a longitudinal plasmonic resonance wavelength of 650 nm were synthesized to overlap with the Fluorescent dye IR650 excitation and emission.^28^ Subsequently, the AuNR@Ag nanocuboids were coated with an ∼3 nm thick siloxane copolymer layer and BSA-conjugated IR650 and streptavidin (Figure S2).

**Figure 2:**
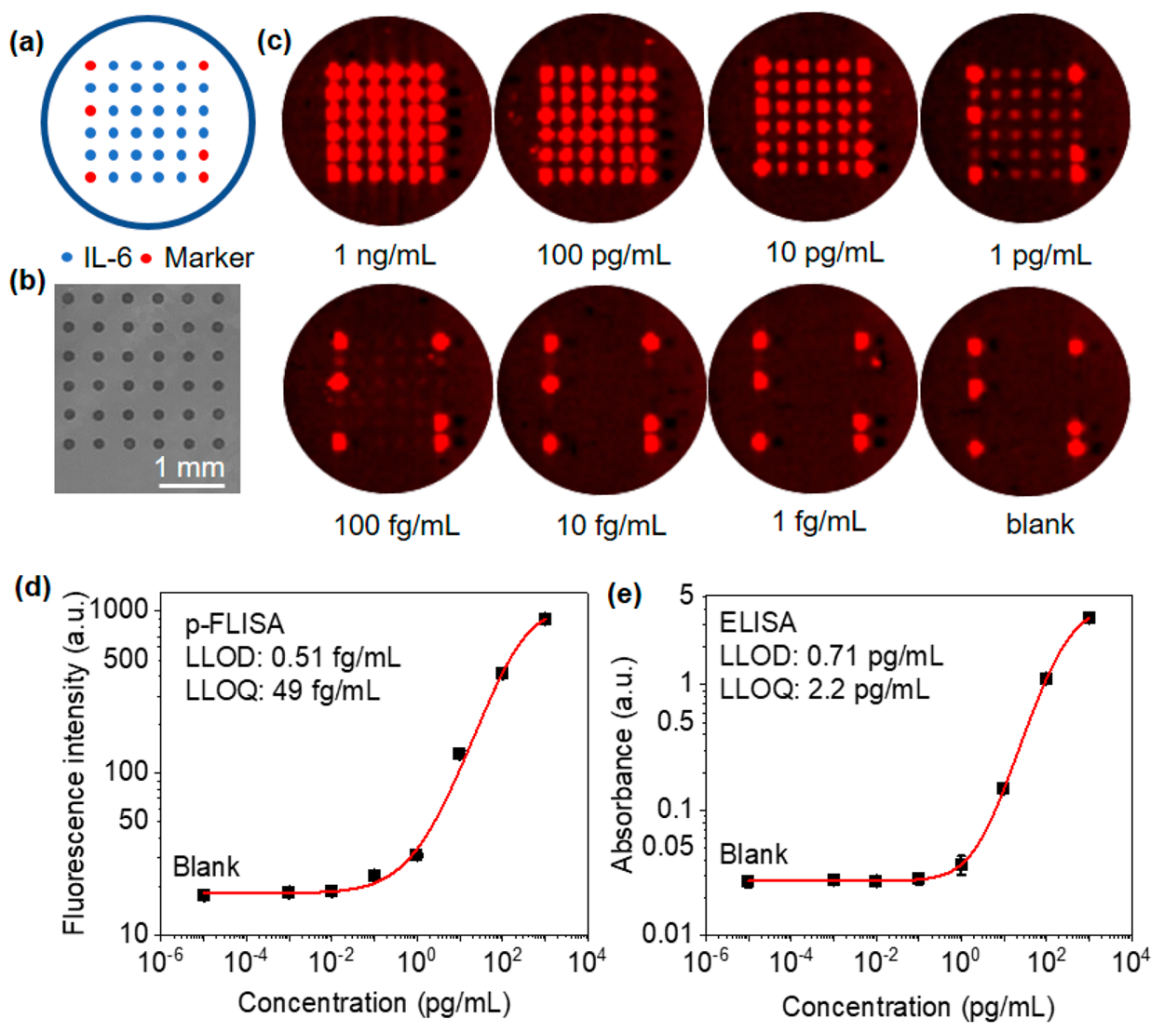
Buoyant sensor with printed antibody microarray. (a) Microarray design with the IL-6 capture antibody microdots and positive control markers. (b) Optical image of the printed antibody microarray. (c) Fluorescence intensity images of the sensors exposed to varying IL-6 concentrations following pFLISA. (d) IL-6 dose-dependent fluorescence intensity on the buoyant sensors following pFLISA. (e) IL-6 dose-dependent absorbance intensity following standard ELISA.

Following pFLISA, we collected the fluorescence images of the microarray on the buoyant sensors after exposing them to changing concentrations of IL-6 for 2 h. The blank control represents the biosensor exposed to the reagent diluent without IL-6. The microdots located at different regions show uniform fluorescence intensity, supporting the capability for spatially multiplexed quantification. The fluorescent intensity increases with the increased IL-6 concentration, while the positive control spots consistently show reliable high fluorescent intensity (Figure 2c). Fluorescence intensity averaged individual microdots (N = 3) was used for the quantification of IL-6 concentration (Figure 2d). Calibration curves were fitted with a five-parameter-logistic method. The low limit of detection (LLOD, defined as mean + 3σ of the blank) and the low limit of quantification (LLOQ, defined as mean + 10σ of the blank) were determined to be 0.51 and 49 fg/mL for IL-6, respectively. Magnified fluorescence images and calibration curve showed that the fluorescence intensity corresponding to 100 fg/mL of IL-6 is much higher than the blank (Figure S3). In addition, the LLOQ obtained from 3 microdots of one sensor is similar to that from 9 microdots of 3 sensors, indicating the high batch-to-batch consistency (Figure S4). In addition to LLOD and LLOQ, we calculated the mean relative error to be less than ±8% and the coefficient of variation to be less than 24% at the concentration range from 0.1 pg/mL to 100 pg/mL, based on the validation method recommended previously^29^ (Figure S5). For comparison, the ELISA provides the LLOD and LLOQ of 0.71 and 2.2 pg/mL, 1392- and 45-fold higher than those of pFLISA (Figure 2e). These results confirm the higher sensitivity of buoyant sensors based on pFLISA compared to ELISA.

Temporal resolution is important to reveal dynamic changes in the concentration of cytokines in biofluids. Standard ELISA typically involves 2 h of sample incubation to achieve the desired sensitivity. Here, we examined the effect of sampling time on sensitivity by reducing the sampling duration from 2 h to 15 min. In these experiments, the sensors floated on the culture media spiked with varying concentrations of IL-6 and were kept at 37 °C in an incubator, consistent with cell culture experimental conditions. Figure 3a shows the calibration curve of averaged fluorescent intensity as a function of IL-6 concentrations from 10 ng/mL to 1 fg/mL with a 2 h sampling time. The LLOD and LLOQ were calculated to be 1.4 and 29 fg/mL. As expected, shorter sampling times decreased the fluorescence intensity and increased the LLOD and LLOQ following the reduced number of captured IL-6 targets (Figures 3b and S6). With 15-min sampling time, the LLOD and LLOQ increased to 3.0 fg/mL and 69 fg/mL, respectively, twice as high as those with 2-h sampling but still much lower than those provided by ELISA with 2-h sampling.

**Figure 3:**
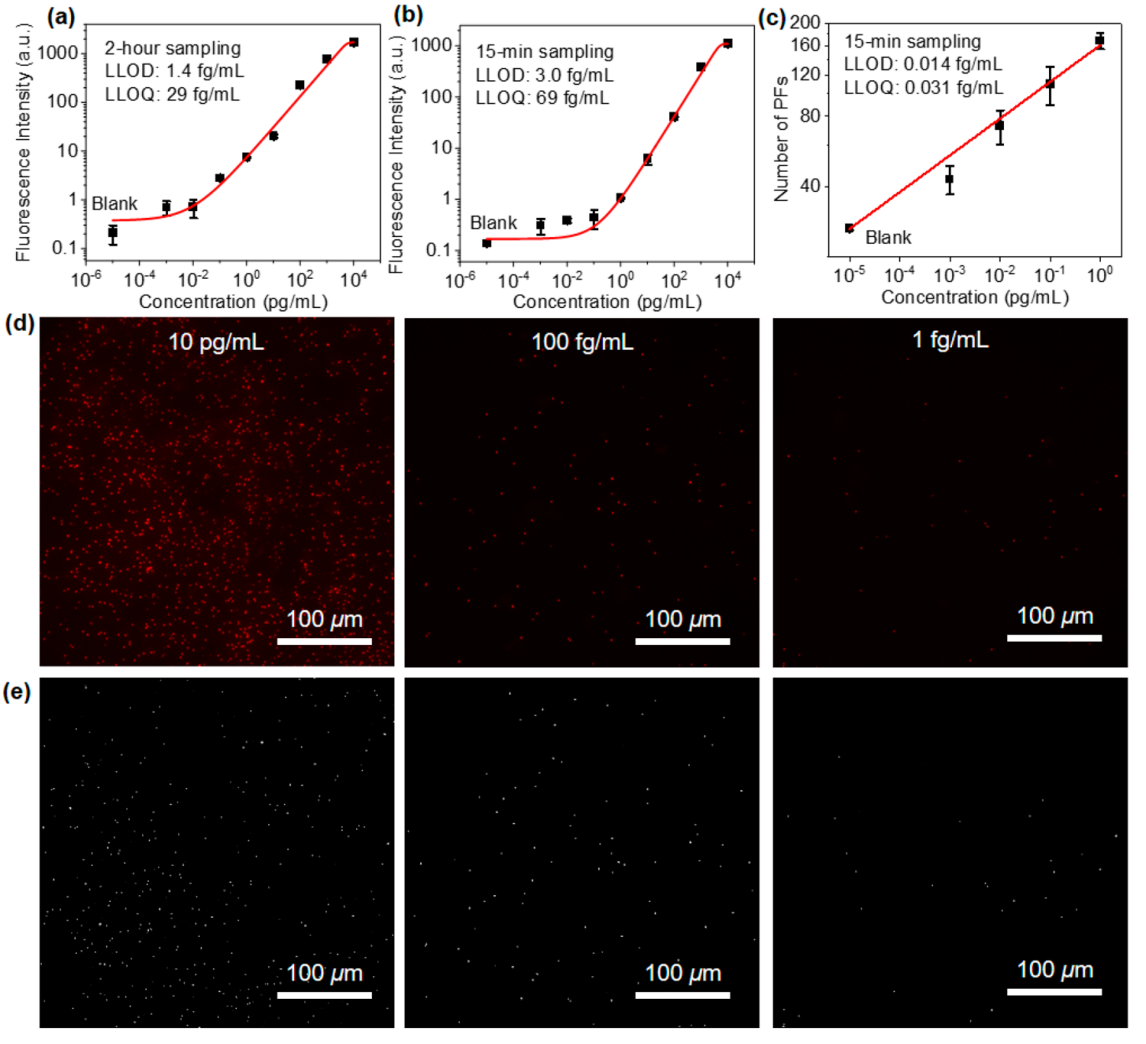
Effect of the sampling time. IL-6 dose-dependent fluorescence intensity on the buoyant sensors following pFLISA with (a) 2 h and (b) 15 min sampling time. (c) IL-6 dose-dependent fluorescence digital counting following pFLISA with 15-min sampling time. (d) Representative fluorescence microscopy images and (e) Gaussian blur processed images of the buoyant sensors exposed to different IL-6 concentrations used for digital analysis.

Immunoassays based on digital imaging technologies can provide higher sensitivity than ensemble average measurements by counting the labels linked to the binding of biomolecule analytes.^30−34^ To further decrease the LLOD, we performed digital counting of PF nanolabels from fluorescence microscope images collected at the center of the microdots corresponding to the 15 min sampling (Figures 3d, S7a, and S8a). Figure 3d shows representative fluorescence images with a field of view 375 μm × 375 μm corresponding to IL-6 concentrations from 10 pg/mL to 1 fg/mL. With decreasing IL-6 concentration, decreased numbers of PF nanolabels were observed. After processing the images by first Gaussian blur, where the high-frequency noise was filtered, the bright individual dots could be visualized and counted (Figures 3e, S7b, and S8b). This method was sensitive at low concentrations while saturated at concentrations of >100 pg/mL (Figure S9). A calibration curve was fitted using a linear function, and the LLOD and LLOQ were calculated to be 0.014 and 0.031 fg/mL, respectively (Figure 3c). The fitting quality can be improved after removing the blank data point from the calibration curve, although this slightly increased the LLOD and LLOQ to 0.096 and 0.18 fg/mL (Figure S10). This confirms that the digital counting approach can further decrease the LLOD and LLOQ and expand the dynamic range by ∼3 orders of magnitude for the quantification of cytokines based on pFLISA.

Next, we demonstrate the spatial multiplexing capability of the buoyant sensors for the simultaneous quantification of IL-6 and TNF-α. To examine the possible cross-reactivity, we exposed the multiplexed sensor to a series of varying concentrations of IL-6 and TNF-α spiked in culture media and quantified the fluorescence intensity following pFLISA. Figure 4a shows the fluorescence intensity of IL-6 antibody microdots exposed to varying concentrations of IL-6 and TNF-α. The fluorescence intensity resulting from the binding of TNF-α at all concentrations is low. The fluorescence intensity corresponding to increasing concentration of IL-6 shows a consistent trend with singleplex sensors and only a slight increase in the LLOQ from 69 to 140 fg/mL. Similarly, the fluorescence intensity of TNF-α antibody microdots was quantified following the exposure of varying concentrations of IL-6 and TNF-α (Figure 4b). The LLOD and LLOQ for TNF-α were calculated to be 21 fg/mL and 0.37 pg/mL, respectively. In comparison, the standard ELISA provides the higher LLOD and LLOQ of 0.33 and 1.1 pg/mL (Figure 4c). The fluorescent intensity resulting from the binding of IL-6 to the TNF-α antibody microdots is comparable to that from the binding of TNF-α at a low concentration of 0.1 pg/mL. We further validated the multiplexing capability with four cytokine targets, including human IL-6, human TNF-α, mouse IL-6, and mouse TNF-α, to challenge the cross-reactivity between different species. Our results show that mouse IL-6 and TNF-α antibodies do not exhibit cross-reactivity with human IL-6 and TNF-α, consistent with the statement from the vendor (Figure S11). The LLOQ for mouse IL-6 and TNF-α was calculated to be 29 fg/mL and 0.49 pg/mL, respectively.

**Figure 4:**
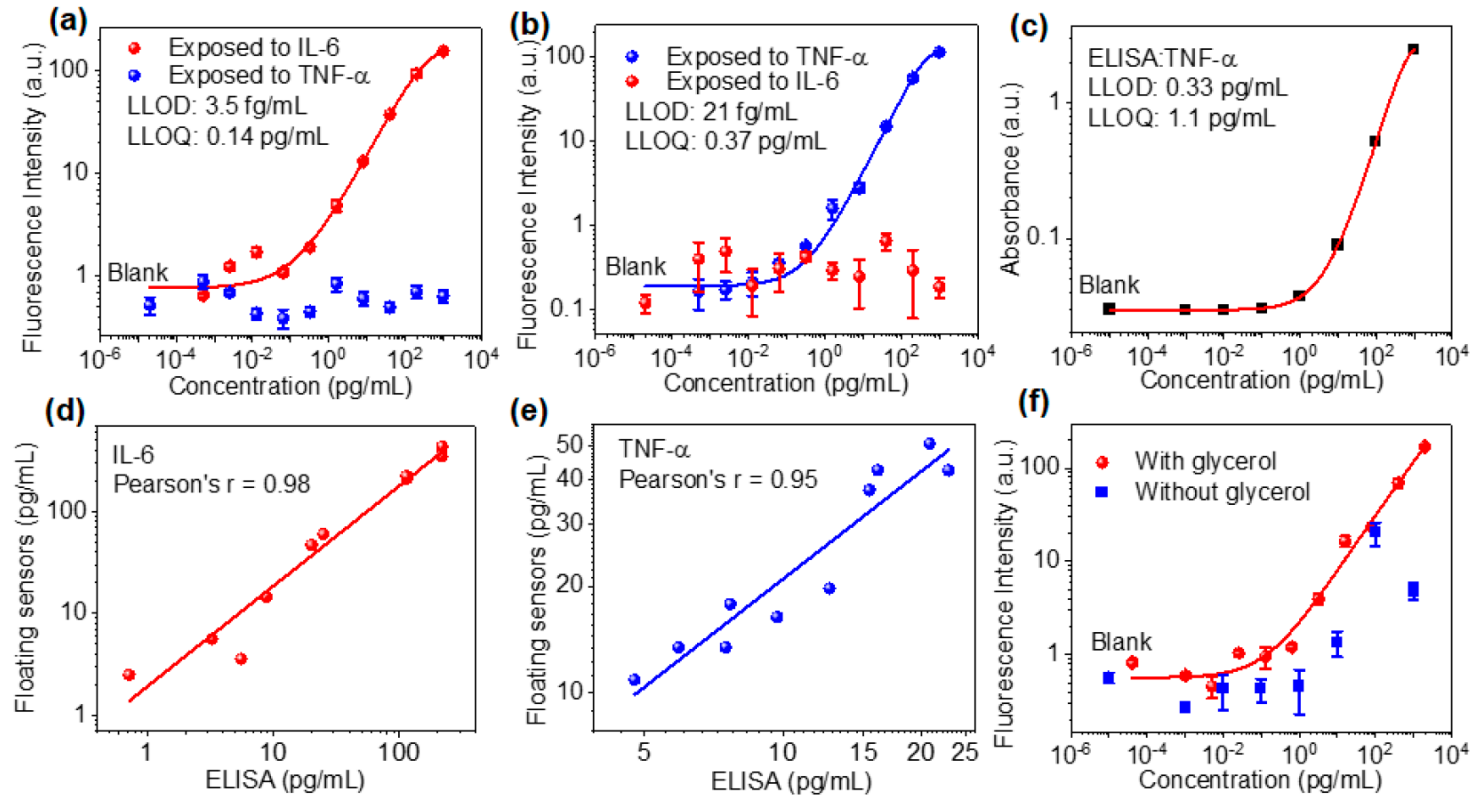
Multiplexed quantification of protein analytes and preservation of the sensors. (a) Fluorescence intensities from IL-6 capture antibody arrays after exposure to different concentrations of IL-6 and TNF-α. (b) Fluorescence intensities from TNF-α capture antibody arrays after exposure to different concentrations of TNF-α and IL-6. (c) TNF-α calibration curve using standard ELISA. (d) Linear regression plot of IL-6 concentration in cell culture medium samples using standard ELISA and floating multiplex sensor. (e) Linear regression plot of TNF-α concentration in cell culture medium samples using standard ELISA and floating multiplex sensor. (f) Fluorescence intensities resulting from IL-6 at different concentrations measured from the sensor with and without glycerol preservation 24 h after the sensor preparation.

For performance validation, we simultaneously quantified human IL-6 and TNF-α in cell culture media collected from macrophage cell cultures using multiplexed buoyant sensors and standard ELISA. Linear regression between the ELISA and multiplex sensor quantified IL-6 and TNF-α concentrations shows high correlations with Pearson’s r values of 0.98 and 0.95, respectively (Figure 4d,e). We assessed and validated the sensor accuracy through spike-and-recovery and linearity-of-dilution experiments and found recovery rates in the 85% to 105% range (Tables S1 and S2). These results confirm the effectiveness of using the pristine culture medium for calibration to quantify the analyte in the macrophage culture supernatant. To improve the stability of printed antibodies, a thin layer of glycerol was spin-coated on the PS surface after printing. The sensors show only a slight degradation in sensitivity after storage at room temperature for 24 h compared with the freshly prepared sensors. Without this preservation strategy, the sensors exhibited significant degradation in sensitivity and a detectable fluorescence signal was observed only at high concentrations of IL-6 (>10 pg/mL) due to the denaturation of the immobilized capture antibody (Figure 4f). Storing the glycerol-coated sensors at 4 °C improves the antibody stability. The sensors showed similar sensitivity after 1 day of storage at 4 °C and increased LLOQ from 29 to 95 fg/mL after 3 days (Figure S12). Alternatively, other preservation strategies, such as molecular encapsulation and metal–organic framework coating, can also be applied to further improve antibody stability.^26,35^

As a proof of concept, we demonstrated the use of our buoyant sensors for in situ monitoring of IL-6 and TNF-α in macrophage cell cultures. We utilized a human leukemia monocytic cell line (THP-1) in these experiments, which is a well-established *in vitro* cell model to study monocyte and macrophage functions.^36^ Phorbol 12-myristate 13-acetate (PMA, 50 ng/mL) was used to differentiate the human THP-1 monocytes into macrophages (M0; Figure 5a). The second cell group is M1 macrophages classically activated with lipopolysaccharide (LPS) and interferon gamma (IFN-γ).^4^ The sensor components were sterilized with 75% ethanol to avoid cell culture contamination. We evaluated the sensor biocompatibility by live/dead cell assay after floating the sensors on the surface of M0 and M1 cell cultures for 7 h. Figure 5b shows overlaid fluorescence images of cells after live/dead staining, with green indicating live cells and red indicating dead cells. The cell morphology and dimensions in the standard cultures are consistent with those with sensors (Figure S13). The cell viability of both M0 and M1 in all cases is higher than 96% (Figure 5c). The sensor biocompatibility was further confirmed by the similar cell viability after 3 days of sensor incubation in cell culture as the control (Figure S14).

**Figure 5:**
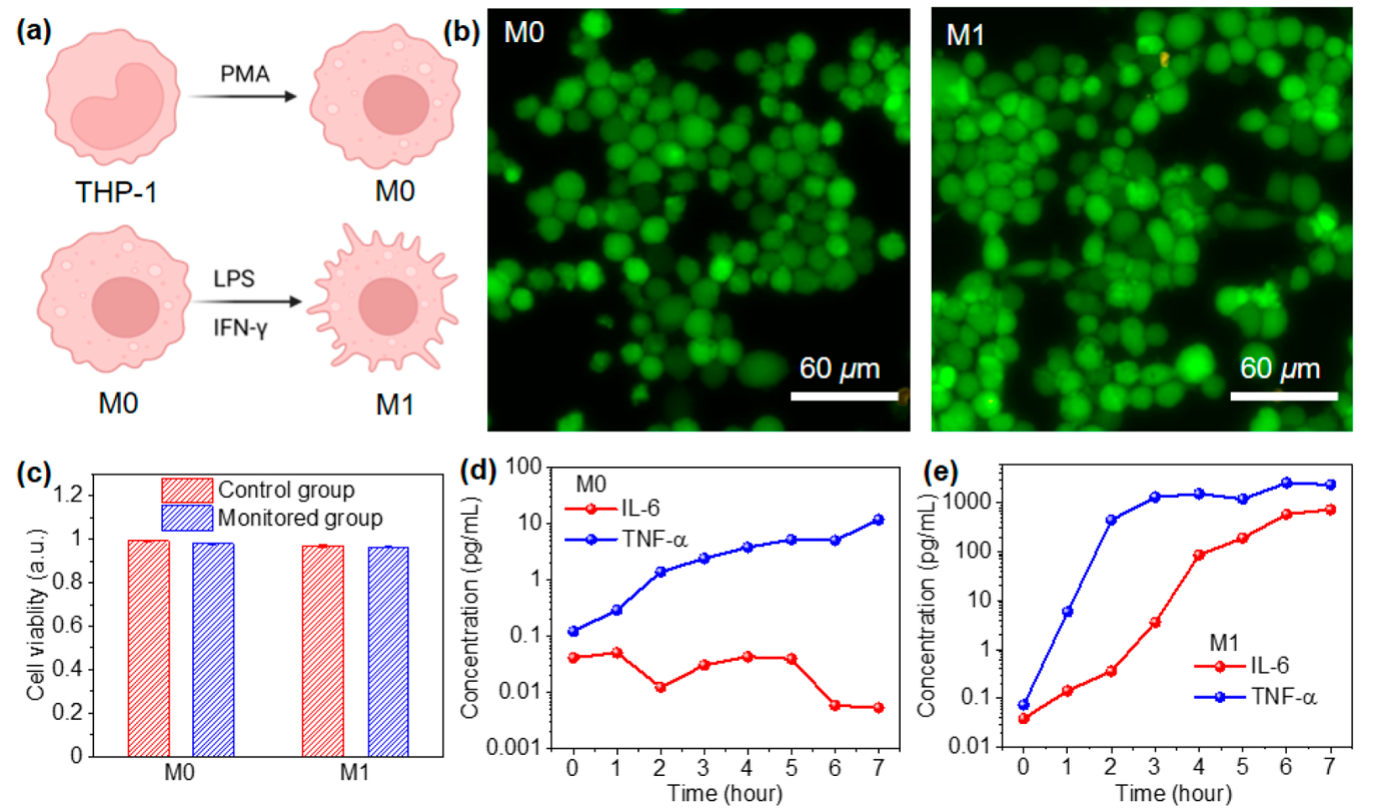
In-situ monitoring of cytokines in macrophage cell cultures. (a) Schematic of macrophage differentiation. (b) Overlaid fluorescence images of M0 and M1 macrophages after exposure to buoyant sensors with green labeling live and red labeling dead cells. (c) Cell viability of macrophages with and without exposure to buoyant sensors. Concentrations of IL-6 and TNF-α secreted by (d) M0 and (e) M1 macrophages quantified by multiplexed buoyant sensors.

Macrophage M1 polarization leads to increased concentrations of proinflammatory cytokines, including IL-6 and TNF-α.^4,5^ However, the temporal concentration changes could only be measured every several hours from different cell populations with replicated cell culture conditions because of limitations with the existing bioanalytical approaches, including ELISA. In contrast, our approach can simultaneously quantify the secreted cytokine concentrations with a temporal resolution of 1 h or a shorter time interval from the same cell populations. After immobilizing M0 for 24 h, the cell culture media with PMA were changed with new media with and without LPS and IFN-γ. Subsequently, 8 buoyant sensors were introduced on the culture media surface every hour and incubated for 15 min, and the dynamic changes in the IL-6 and TNF-α levels were monitored over ∼7 h. In the M0 macrophage cell culture, the IL-6 concentration stayed below 0.1 pg/mL and slightly decreased below 10 fg/mL after 5 h, while the TNF-α concentration gradually increased by 2 orders of magnitude from 0.12 pg/mL to 12 pg/mL in 7 h (Figure 5d). The results indicate a low level of pro-inflammatory cytokine secretion in M0 macrophages. In contrast, in the presence of LPS and IFN-γ, macrophages secreted high levels of proinflammatory cytokines (Figure 5e). The IL-6 concentration increased by more than 4 orders of magnitude from 39 fg/mL to 590 pg/mL in 6 h. The TNF-α concentration increased from 74 fg/mL to 1.3 ng/mL in 3 h and reached a plateau. It is important to note that the initial low concentrations of IL-6 and TNF-α are not detectable and quantifiable with ELISA.

The introduction of a nonperturbing biosensor enables the quantification of protein analytes at attomolar concentrations with high temporal resolution. The biosensor can interface with the biofluids and capture target biomarkers *in situ* without disrupting biological systems. The biosensor with antibody arrays enables the simultaneous quantification of multiple cytokines. With digital counting, the LLOD for IL-6 was below 1 aM and 246-fold lower than the LLOD defined by the average fluorescence intensity analysis. The record high sensitivity reported here mainly results from the quantification approach via digital counting ultrabright nanolabels, high binding properties of the Gamma irradiated PS surface, and strong affinity between the antibody and target proteins. Such a strong affinity enables the capture of target proteins at very low concentrations while making their continuous monitoring challenging without a refreshing mechanism. Our ongoing efforts focus on refreshing the binding sites to release captured molecules for continuous monitoring. With its attomolar sensitivity and multiplexing capabilities, this user-friendly biosensor offers a valuable tool for *in situ* analysis of protein analytes in various biofluids and tissues.

## Supporting Information Available

Experimental section including materials, sensor fabrication, human IL-6 and TNF-α ELISA and pFLISA, and macrophage culture details; optical and fluorescence images of PS films; TEM image of PF nanolabels; fluorescence sensor images and dose-dependent fluorescence intensity; mean relative error and coefficient of variation analysis; fluorescence microscopy images of buoyant sensors before and after Gaussian blur; fluorescence images of cells after live/dead staining; and recovery rate calculations (PDF)
