# Supplementary material for: Ultrasensitive, Multiplexed Buoyant Sensor for Monitoring Cytokines in Biofluids
Source: Nano Lett. 2023 Nov 3;23(22):10171–8. doi: 10.1021/acs.nanolett.3c02516 (PMC10863391; doi:10.1021/acs.nanolett.3c02516)
Supplement: Supplementary file 1 — nl3c02516_si_001.pdf [file nl3c02516_si_001.pdf]

## Supporting Information

### Ultrasensitive, Multiplexed Buoyant Sensor for Monitoring Cytokines in Biofluids

Heng Guo[a], Rohit Gupta[b], Dhavan Sharma[a], Elizabeth Zhanov[a], Connor Malone[a], Ravi Jada[a], Ying Liu[a], Mayank Garg[a], Srikanth Singamaneni[b], Feng Zhao[a], Limei Tian[a]*

[a]*Department of Biomedical Engineering, Texas A&M University, College Station, TX 77843, United States*

[b]*Department of Mechanical Engineering and Materials Science, Institute of Materials Science and Engineering, Washington University in St. Louis, St. Louis, MO 63130, United States*

*Email: ltian@tamu.edu

## Experimental section

**Materials.** Microplates, capture antibodies, biotinylated detection antibodies, IL-6, TNF-α, horseradish peroxidase (HRP)-labeled streptavidin, color reagents, and stop solution (2N $H_2SO_4$) were purchased from R&D Systems. Phosphate-buffered saline (PBS, 10X) was purchased from Invitrogen. BSA was purchased from Sigma-Aldrich. Tween-20 was purchased from Fisher Scientific. Dow SYLGARD™ 184 Silicone was purchased from Ellsworth Adhesives. Polystyrene film was purchased from Goodfellow. THP-1 cells and Roswell Park Memorial Institute (RPMI)-1640 media were purchased from ATCC. The fetal bovine serum (FBS) was purchased from R&D. Penicillin/streptomycin (pen-strep) was purchased from Thermo Fisher. PMA and LPS were purchased from Sigma Aldrich. IFN-γ was purchased from Cell Signaling Technology. Plasmonic-fluor nanolabels (PF650TM ultrabright fluor), synthesized according to our previous report[14], were obtained from Auragent Bioscience LLC (St. Louis, USA).

**Sensor fabrication.** For PDMS preparation, SYLGARD™ 184 Silicone Elastomer base and curing agent were mixed at a 10:1 ratio, degassed, transferred into Petri dishes to form a 600-$\mu$m thick sheet, and then kept for curing at 60°C for 12 hours. Next, the PDMS sheet was cut into circular disks of 5 mm in diameter with a metal disc cutter. The PS was treated with Gamma irradiation and cut with picosecond-fast laser pulses (frequency: 300 kHz; power: 2 W; mark speed: 400 mm/s; repetitions: 4). The sensors prepared with pristine PS were found to have lower sensitivity than those prepared with Gamma-irradiated PS (Figure S15). The PDMS and PS were sterilized with 75% ethanol and then assembled using a vacuum pick-up tool to form buoyant sensors. Capture antibodies (120 $\mu$g/mL in 1X PBS for IL-6 and 240 $\mu$g/mL in 1X PBS for TNF-α) were printed on the PS surface using an electromagnetic droplet jetting nozzle and incubated for 30 mins at room temperature. Next, the buoyant sensors were rinsed with washing buffer (0.05% Tween-20 in 1X PBS) 3 times and blocked with 3 wt% BSA in 1× PBS for one hour.

**Human IL-6 and TNF-α ELISA.** The ELISA procedure follows the optimized protocol provided by the vendor (R&D Systems). A 96-well microplate was first coated with capture antibody (2 $\mu$g/mL in 1X PBS for IL-6 and 4 $\mu$g/mL in 1X PBS for TNF-α) through overnight incubation. Next, the plate was rinsed with washing buffer (0.05% Tween-20 in 1X PBS) 3 times and blocked with 3 wt% BSA in 1X PBS for one hour. After rinsing, varying concentrations of IL-6 and TNF-α spiked in reagent diluent (1wt% BSA in 1X PBS) or cell culture medium (RPMI 1640 medium supplemented with 10% FBS and 1% pen-strep), and the samples collected from cell cultures were added to the microplate wells. After 2 hours of incubation, the microplate was rinsed and incubated with the biotinylated detection antibody (50 ng/mL in reagent diluent for both IL-6 and TNF-α) for 2 hours. Subsequently, the microplate was rinsed and incubated with HRP-labelled streptavidin for 20 minutes. After rinsing, the substrate solution (1:1 mixture of $H_2O_2$ and tetramethylbenzidine) was added to the microplate to allow an enzymatic reaction followed by a stop solution after 20 minutes. All the incubation steps were at room temperature without disturbance. The optical density was immediately determined using a microplate reader (Tecan Infinity M Nano Plus).

**Human IL-6 and TNF-α pFLISA.**

The sensors were exposed to 100 $\mu$L varying concentrations of IL-6 and TNF-α spiked in reagent diluent (1wt% BSA in 1X phosphate-buffered saline) or cell culture media in a floating

format with a sampling duration from 15 minutes to 2 hours at 37°C. Subsequently, the buoyant sensors were transferred into a 96-well microplate and rinsed with wash buffer (0.05% Tween-20 in 1X PBS) 3 times. The sensors were then incubated with the biotinylated detection antibody (50 ng/mL in reagent diluent for both IL-6 and TNF-α) for 2 hours, followed by the wash buffer rinsing 3 times, 100 $\mu$L PF nanolabel solution (optical density ~0.5) was added and incubated for 30 minutes, followed by rinsing 3 times. Finally, the sensors were imaged using LI-COR fluorescence imager and BioTek Cytation 5 Cell Imaging Multi-Mode Reader. For imaging analysis, at least three microdots were used to obtain mean fluorescent intensity and nanolabel numbers and corresponding standard deviations. To monitor IL-6 and TNF-α in macrophage culture, the sensors were introduced to the surface of culture media at different times and floated for 15 minutes to capture the targets. All other steps follow the same for IL-6 and TNF-α quantification. To validate the sensor accuracy, we performed spike-and-recovery and linearity-of-dilution experiments. In spike-and-recovery experiments, 90 μL macrophage culture supernatant was spiked with 10 μL pristine cell culture medium (RPMI-1640 with 10% FBS and 1% pen-strep) containing 0, 250 pg/mL, 500 pg/mL, and 1 ng/mL IL-6. Standard IL-6 in pristine culture medium at concentrations of 0, 25 pg/mL, 50 pg/mL, and 100 pg/mL served as controls. The IL-6 concentrations in the controls and samples were quantified using our sensors, and percentage recovery was calculated using the equation: Recovery rate = [(observed sample spike concentration - observed sample initial concentration) / expected concentration] x 100% (Table S1). In linearity-of-dilution experiments, macrophage culture supernatant was diluted with pristine culture medium at different dilution factors of 1:2, 1:4, and 1:8 and then quantified with our sensors. Percentage recovery was calculated using the equation: Recovery rate = (observed sample concentration / expected concentration) x 100% (Table S2).

**Macrophage culture.** The THP-1 cell culture follows the previous protocol[5]. RPMI-1640 media supplemented with 10% FBS and 1% pen-strep was used to culture THP-1 cells. For differentiation into macrophages, the THP-1 cells cultured in the 24-well microplate with a cell density of 200,000 cells/cm$^2$ were treated with the culture medium containing 50 ng/mL PMA. It took 24 hours for the THP-1 cells to differentiate and adhere to the microplate. The fresh cell culture media (RPMI 1640 medium supplemented with 10% FBS and 1% pen-strep) with 100 ng/mL LPS and 15 ng/mL IFN-γ were added to classically activate M0 into M1. For live/dead viability staining, the cells were rinsed twice with sterile Dulbecco's PBS (DPBS) to thoroughly remove the culture medium and then immersed in the mixture of Calcein AM (2 $\mu$M in DPBS) and Ethidium Homodimer-1 (4 $\mu$M in DPBS) for 30 minutes at room temperature. The cells were imaged under a fluorescence microscope (Zeiss Axio Observer 3) with red (ex/em 495 nm/635 nm) and green (ex/em 495 nm/515 nm) channels to observe dead and live cells, respectively.

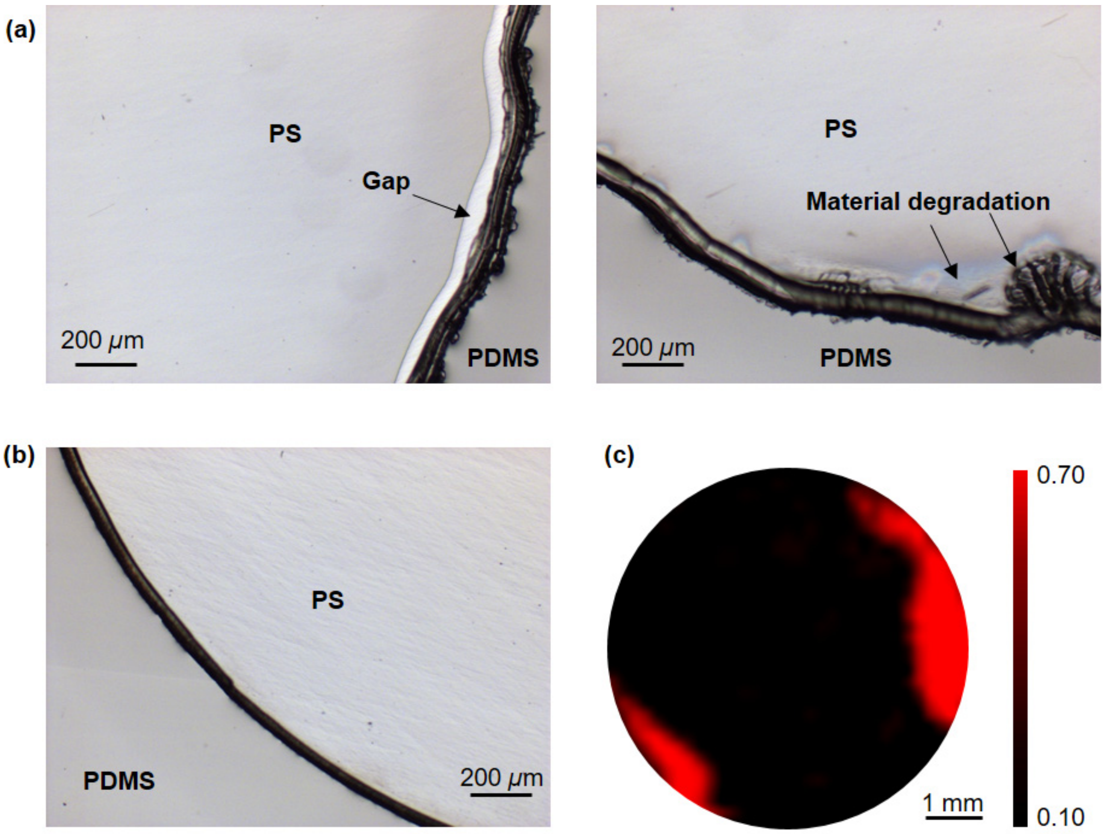


**Figure S1. PS film cut with different methods.** (a) Optical images of PS film cut by $CO_2$ laser laminated on PDMS surface. The edge of circular PS film shows plastic deformation and materials degradation. (b) Optical image of PS cut by picosecond-fast laser pulses laminated on PDMS surface. (c) Fluorescence image of a buoyant sensor after pFLISA showing unwanted fluorescence background at the edge of the sensor, resulting from the defects introduced during PS cutting shown in (a).

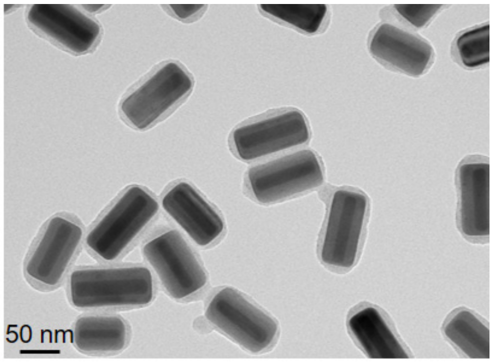


**Figure S2.** TEM image of PF nanolabels.

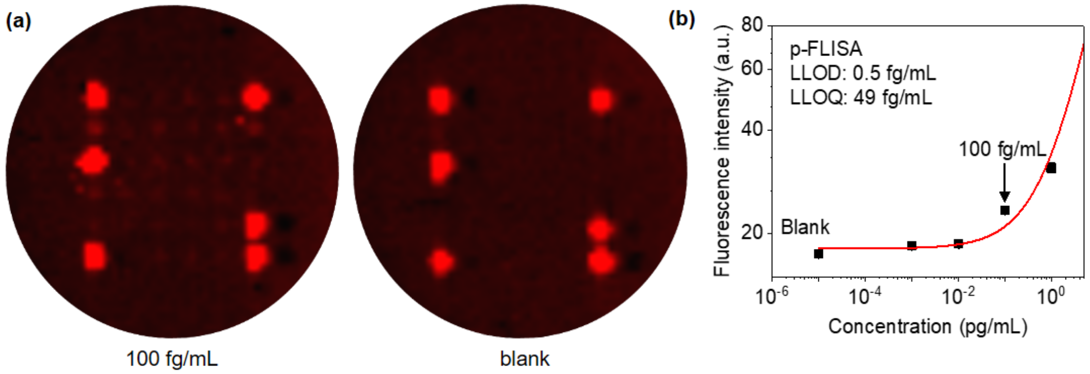


**Figure S3.** (a) Fluorescence intensity images of the sensors exposed to 100 fg/mL of IL-6 and blank (reagent dilute without IL-6) following pFLISA. (b) Zoomed-in IL-6 dose-dependent fluorescence intensity at a low concentration range.

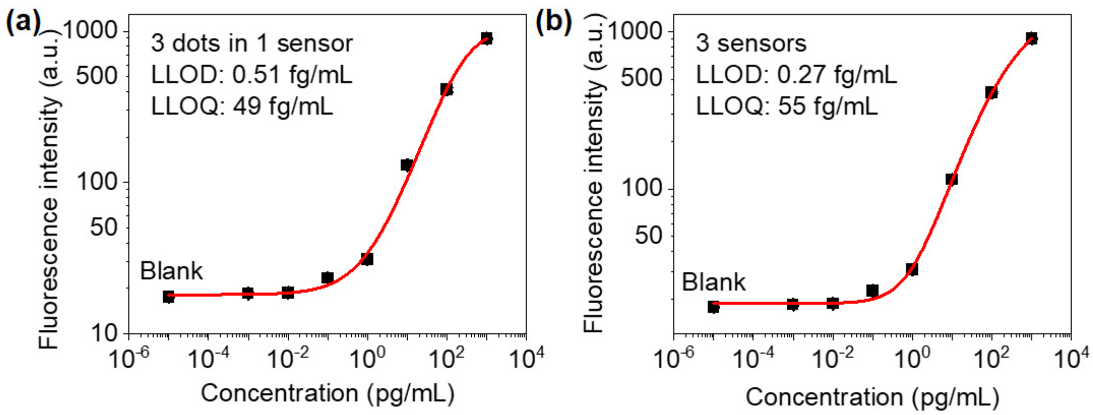


**Figure S4.** (a) IL-6 dose-dependent fluorescence intensity following pFLISA collected from 3 microdots on one sensor. (b) IL-6 dose-dependent fluorescence intensity following pFLISA collected from 9 microdots of 3 sensors (3 microdots in each sensor).

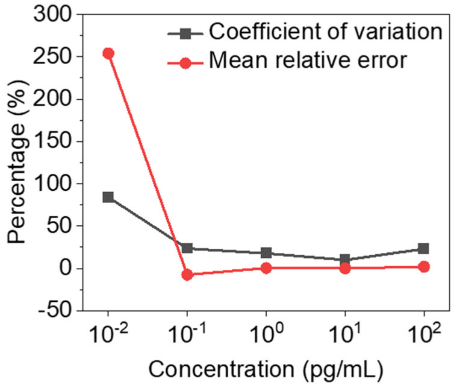


**Figure S5.** Percent mean relative error and coefficient of variation in back-calculated concentrations at the concentration range from 0.1 pg/mL to 100 pg/mL.

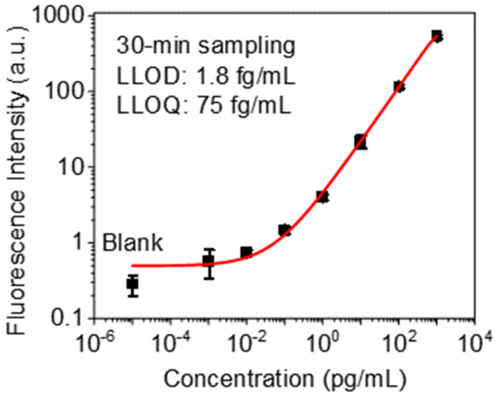


**Figure S6.** IL-6 dose-dependent fluorescence intensity on the buoyant sensors following pFLISA with 30-min sampling time.

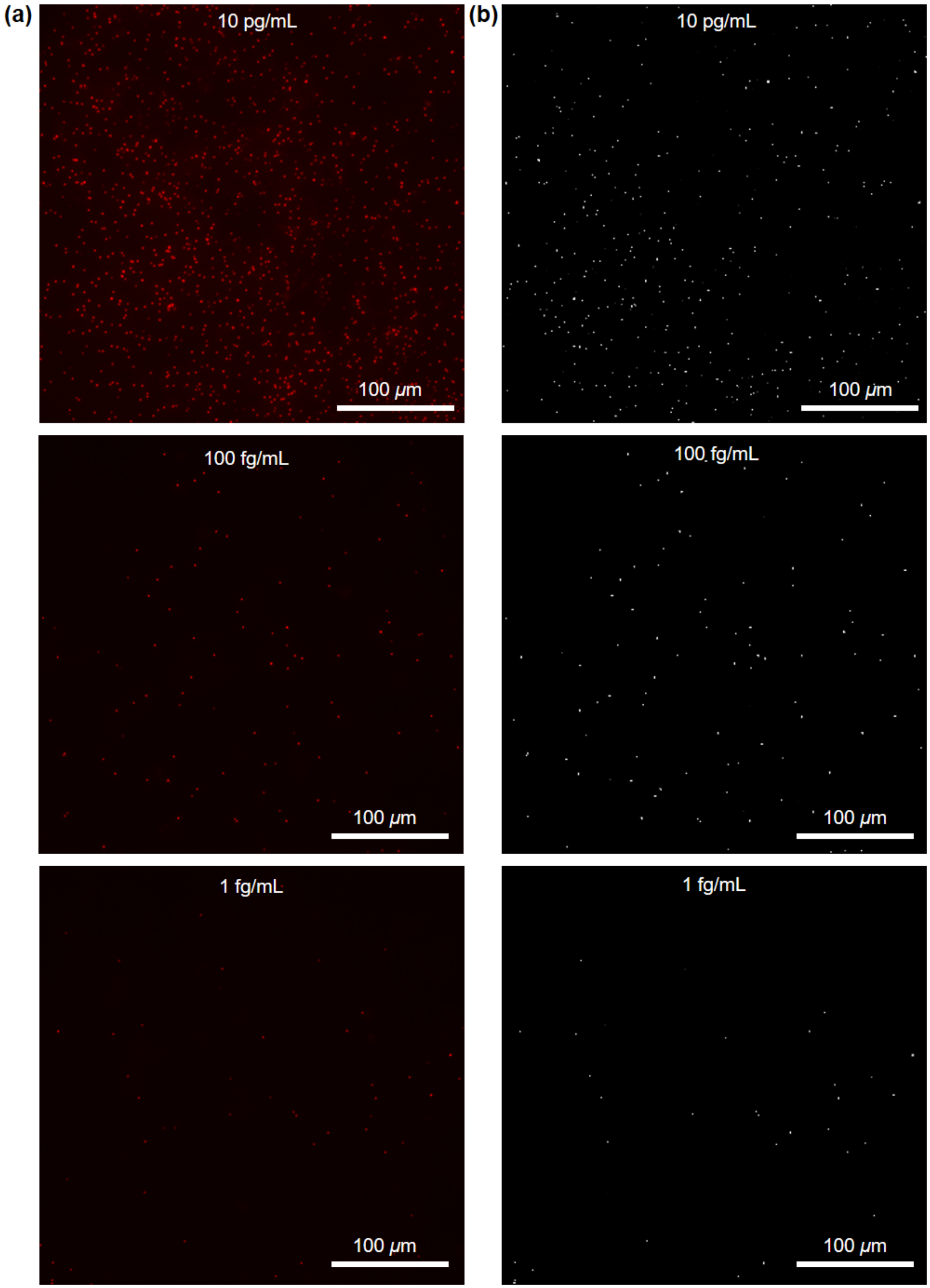


**Figure S7.** (a) Enlarged Fluorescence microscopy images and (b) enlarged Gaussian blur processed images of the buoyant sensors exposed to different IL-6 concentrations shown in Figure 3d-3e.

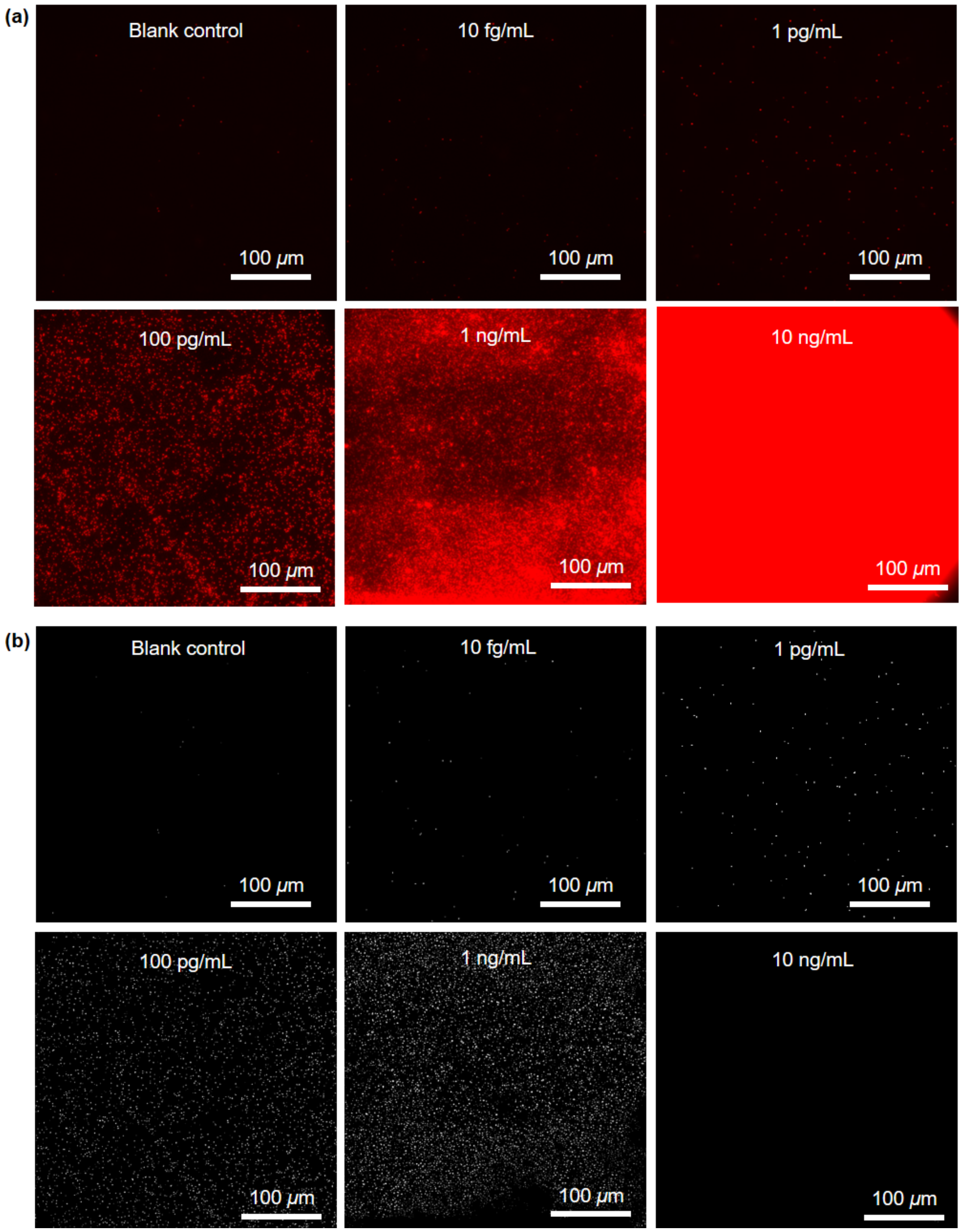


**Figure S8.** (a) Fluorescence microscopy images and (b) Gaussian blur processed images of the buoyant sensors exposed to different IL-6 concentrations used for digital analysis.

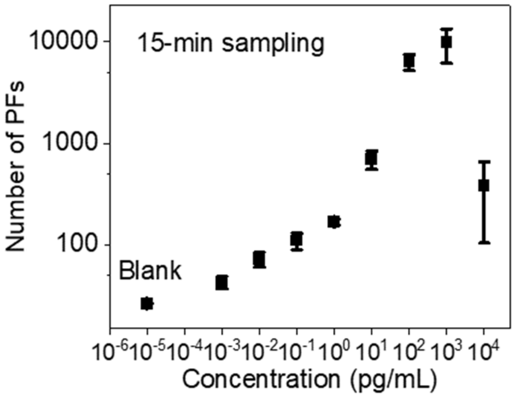


**Figure S9.** IL-6 dose-dependent fluorescence digital counting for an extended range of IL-6 concentrations following pFLISA with 15-min sampling time.

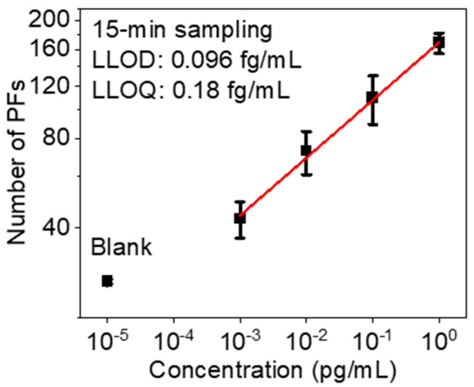


**Figure S10.** IL-6 dose-dependent fluorescence digital counting following pFLISA with 15-min sampling time. A linear fitting was applied after removing the blank data point.

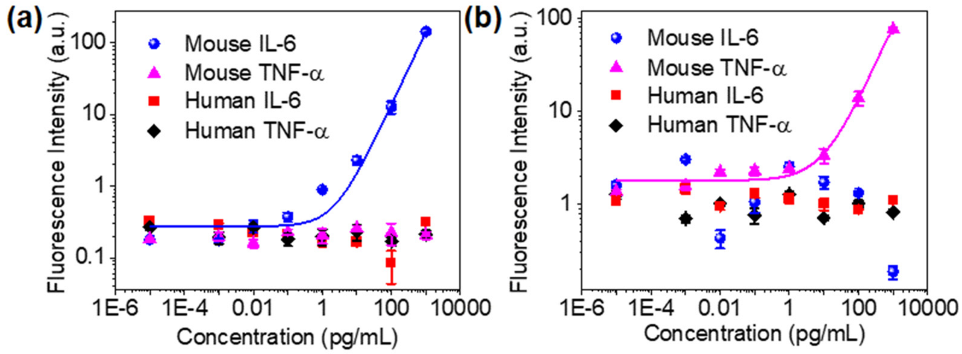


**Figure S11.** (a) Fluorescence intensities from mouse IL-6 capture antibodies after exposure to different concentrations of mouse and human IL-6 and TNF-α. (b) Fluorescence intensities from mouse TNF-α capture antibodies after exposure to different concentrations of mouse and human TNF-α and IL-6.

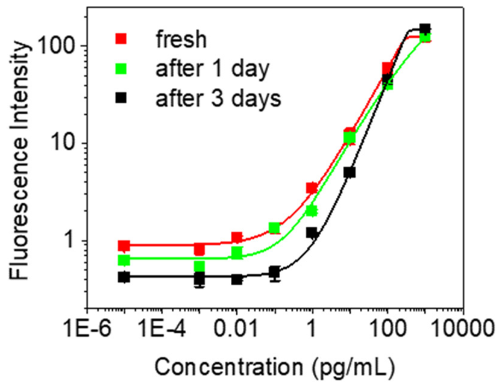


**Figure S12.** Fluorescence intensities resulting from IL-6 at different concentrations measured from the freshly prepared sensors and the glycerol-coated sensors after storage at 4 °C for 1 day and 3 days.

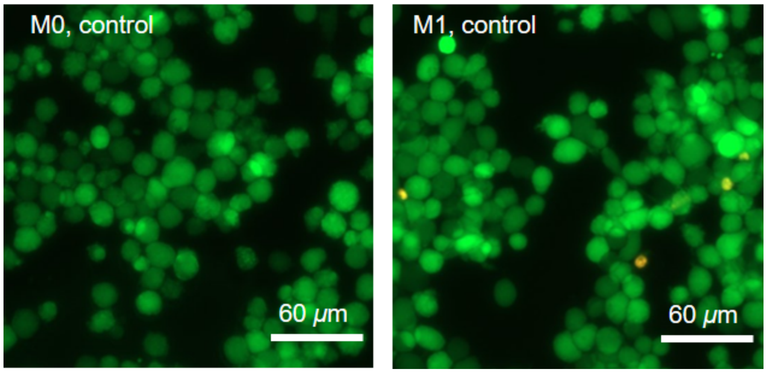


**Figure S13.** Overlaid fluorescence images of M0 and M1 macrophages without exposure to buoyant sensors after live/dead cell staining.

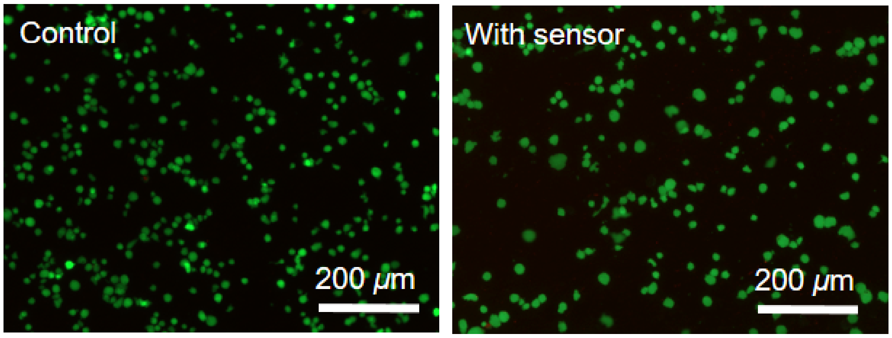


**Figure S14.** Overlaid fluorescence images of cells cultured under standard conditions (control) and with exposure to sensor for 3 days after live/dead cell staining.

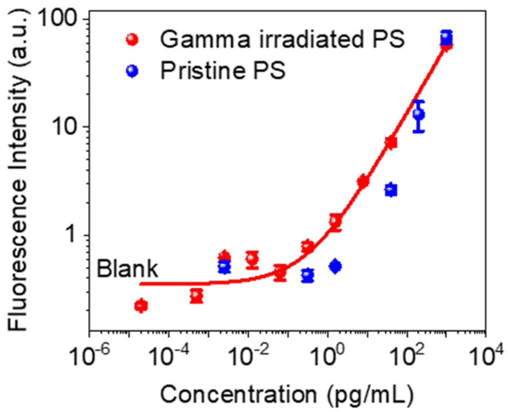


**Figure S15.** TNF-α dose-dependent fluorescence intensity on the buoyant sensors with pristine and Gamma irradiated PS following pFLISA.

**Table S1.** Spike and recovery of IL-6 in macrophage culture supernatant.

| | **No spike** | **Low spike 25 pg/mL** | **Medium spike 50 pg/mL** | **High spike 100 pg/mL** |
|---|---|---|---|---|
| Control: Expected concentration (pg/mL) | 0 | 26.05 | 45.16 | 106.67 |
| Sample: Observed concentration (pg/mL) | 7.17 | 31.26 | 54.20 | 107.12 |
| Recovery rate | NA | 92.5% | 104.2% | 93.7% |

**Table S2.** Linearity-of-dilution results for IL-6 in macrophage culture supernatant.

| Dilution factor (DF) | Observed concentration × DF (pg/mL) | Expected concentration (neat value, pg/mL) | Recovery rate |
|---|---|---|---|
| Neat (undiluted) | 140.60 | 140.60 | 100% |
| 1:2 | 145.23 | | 103.3% |
| 1:4 | 119.29 | | 84.8% |
| 1:8 | 122.69 | | 87.3% |
